# Multi-Level Comparison of Machine Learning Classifiers and Their Performance Metrics

**DOI:** 10.3390/molecules24152811

**Published:** 2019-08-01

**Authors:** Anita Rácz, Dávid Bajusz, Károly Héberger

**Affiliations:** 1 Plasma Chemistry Research Group, Research Centre for Natural Sciences, Hungarian Academy of Sciences, Magyar tudósok krt. 2, H-1117 Budapest, Hungary; 2 Medicinal Chemistry Research Group, Research Centre for Natural Sciences, Hungarian Academy of Sciences, Magyar tudósok krt. 2, H-1117 Budapest, Hungary

**Keywords:** classifiers, performance metrics, ROC, toxicity prediction, ranking, ANOVA, machine learning

## Abstract

Machine learning classification algorithms are widely used for the prediction and classification of the different properties of molecules such as toxicity or biological activity. The prediction of toxic vs. non-toxic molecules is important due to testing on living animals, which has ethical and cost drawbacks as well. The quality of classification models can be determined with several performance parameters. which often give conflicting results. In this study, we performed a multi-level comparison with the use of different performance metrics and machine learning classification methods. Well-established and standardized protocols for the machine learning tasks were used in each case. The comparison was applied to three datasets (acute and aquatic toxicities) and the robust, yet sensitive, sum of ranking differences (SRD) and analysis of variance (ANOVA) were applied for evaluation. The effect of dataset composition (balanced vs. imbalanced) and 2-class vs. multiclass classification scenarios was also studied. Most of the performance metrics are sensitive to dataset composition, especially in 2-class classification problems. The optimal machine learning algorithm also depends significantly on the composition of the dataset.

## 1. Introduction

Model evaluation and selection is an integral, however non-trivial, part of both regression and classification tasks. Especially in the present day, when machine learning [1] and deep learning [2] models are all the rage in drug discovery and related areas, getting proper feedback on model performance is a must: the controversial “black-box” nature [3] of predictive models must be counterbalanced by a thorough understanding from the modeller’s side. This entails proper knowledge of the performance metrics that are used to evaluate classification models and to select the best (or the best few) options. A great number of performance metrics were collected earlier this year by Berrar [4]; his comprehensive work (along with other literature sources) has formed the basis of this study.

In this work, a large number of classification performance metrics from diverse domains are compared in evaluating machine learning-based classification models on three toxicity-related datasets, in 2-class and multiclass scenarios. For the comparison, we apply our proven methodology based on novel and classical chemometric methods, such as sum of ranking differences (SRD) [5] and analysis of variance (ANOVA). For some context on our results for regression/QSAR performance metrics, we direct the reader’s attention to our earlier works [6,7]. A particularly relevant conclusion from these studies is that machine learning methods usually outperform “classic” regression methods; however, principal component regression and partial least squares regression have proven themselves to be the most robust (in terms of the difference between the coefficients of determination for the training set and test set *R^2^* and *Q^2^*, respectively), meaning that special care must be taken with the validation of machine learning models [8]. (Ironically, multiple linear regression (MLR), which produces the largest gap between *R^2^* and *Q^2^*, is still the most popular regression method, based on a recent review of 1533 QSAR publications by Maran et al. [9].)

**Figure 1:**
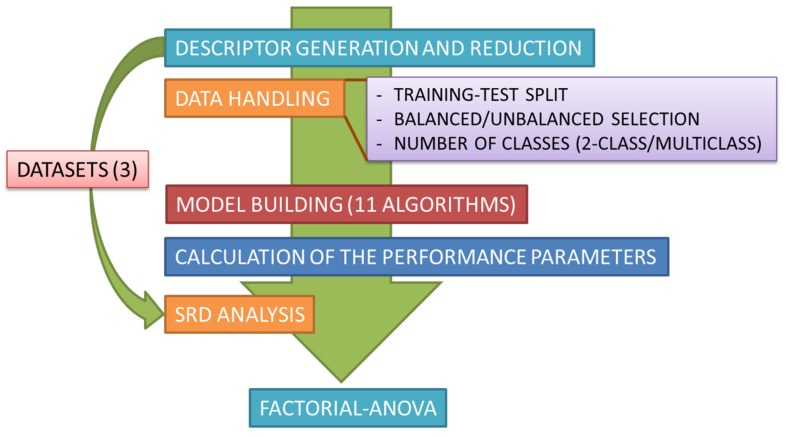
Workflow of the comparative study. Briefly, after descriptor generation and reduction, eleven machine learning methods are applied for model building (for each combination of 2-class/multiclass and balanced/imbalanced cases). After the calculation of the performance parameters, statistical analysis of the results is carried out with sum of ranking differences (SRD) and factorial analysis of variance (ANOVA). The complete process is carried out on three datasets.

In addition to a statistical comparison of 28 performance metrics, we also examine the effect of 2-class vs. multiclass classification, as well as dataset composition (balanced vs. imbalanced) on performance metrics. A comparison of 11 widely-available machine learning classifiers is also reported. The workflow applied for the comparisons is summarized in Figure 1.

## 2. Results and Discussion

### 2.1. Statistical Evaluation of Performance Parameters

**Figure 2:**
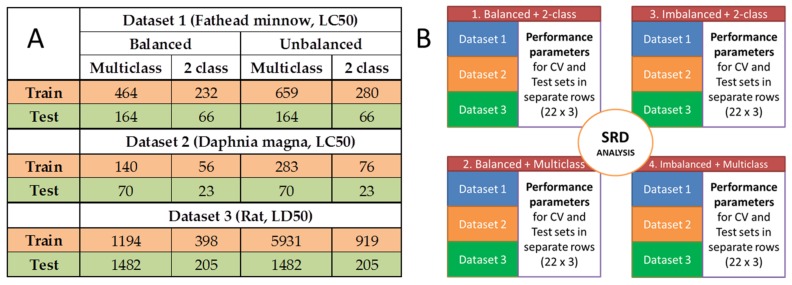
(**A**) Summary of the number of molecules for the three datasets with specific conditions. (**B**) Illustration of merged datasets for the SRD analyses. Datasets 1, 2 and 3 contain the performance parameters of the calculated models. (CV is short for cross-validation.)

The three toxicity datasets (aqueous and acute toxicities, see Section 3.1) were used with the training and test splits indicated at their respective sources. The dataset-specific splits are included in Figure 2A for the balanced and imbalanced designs and the 2-class and multiclass cases.

The number of molecular descriptors (after variable reduction) was always above one thousand, and variable selection was omitted in order to exclude the differences between the applied algorithms and to make the process standardized across different methods. After model building with the eleven machine learning algorithms (see Experimental section), 28 performance parameters were calculated with cross-validation and on the external test sets as well. Then, sum of ranking differences (SRD) was used on the following matrix: the models based on the different algorithms were arranged in rows (22 rows in total, for cross-validation and test validation altogether) and the performance parameters were arranged in columns (28). SRD was used for the three datasets together, with normalization to unit length as data pretreatment. We also tested the effect of balanced vs. imbalanced and 2-class vs. multiclass cases. Thus, the combinations of balanced/imbalanced and 2-class/multiclass versions of the models were handled separately in the SRD analysis. In total, four SRD analyses were carried out on the merged datasets. This part of the analysis is summarized in Figure 2B.

**Figure 3:**
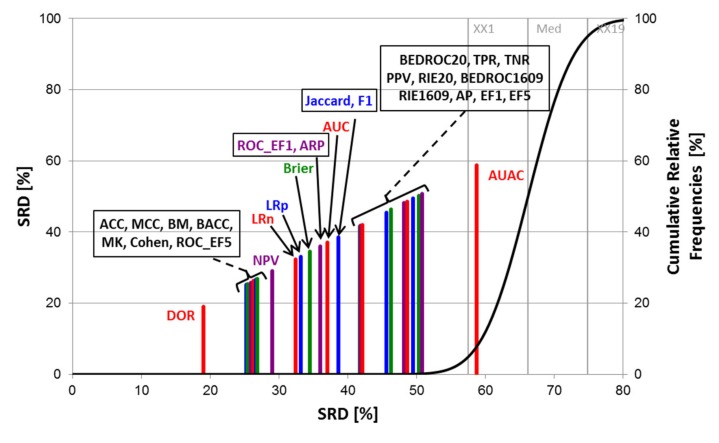
SRD analysis (for the balanced 2-class version). Normalized SRD values are plotted on the X and left Y axes (to make the ordering visually illustrative) ‒ the smaller the better. The abbreviations of the performance metrics can be found in the Section 3.3. The cumulative relative frequencies (right Y axis) correspond to the randomization test (see Section 3.4). Here, the diagnostic odds ratio (DOR) is closest to the reference (smallest SRD value), while AUAC (area under the accumulation curve) overlaps with the cumulative frequency curve, and is therefore statistically indistinguishable from random ranking.

The SRD analyses were carried out with Monte Carlo fivefold cross-validation in ten iterations, and the normalized SRD values were used for the further evaluation of the performance parameters with factorial ANOVA. An example (Balanced 2-class version) of the SRD results can be seen in Figure 3. 

In the ANOVA process, we examined the effect of the following factors on the final results (SRD values): (i) balanced/imbalanced-F1; (ii) 2-class/multiclass-F2; and (iii) performance parameters-F3.

(1) $$SRD=b_{0}+b_{1}F_{1}+b_{2}F_{2}+b_{3}F_{3}+b_{12}F_{1}F_{2}+b_{13}F_{1}F_{3}+b_{23}F_{2}F_{3}+b_{123}F_{1}F_{2}F_{3}$$

For all three factors and all of their combinations, the effects were significant at the α = 0.05 level, meaning that the 2-class vs. multiclass, and the balanced vs. imbalanced distribution between the classes resulted in significantly different SRD values for the performance parameters. On the other hand, the performance metrics were also significantly different, meaning that the final decision in model selection depends strongly on the applied performance metric.

**Figure 4:**
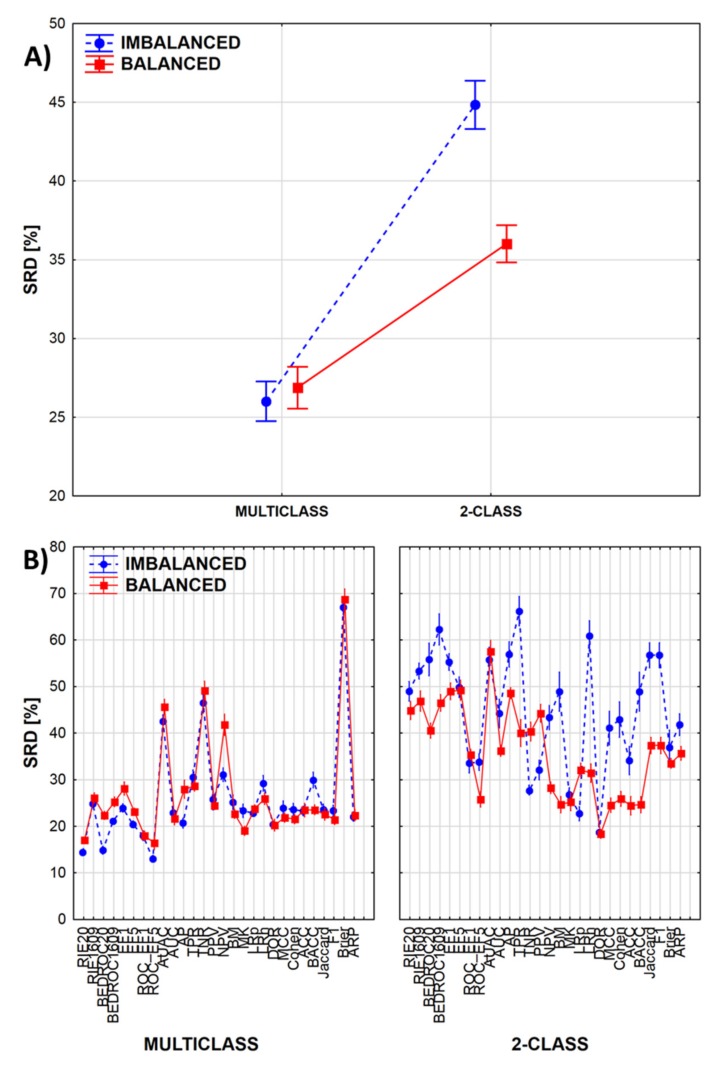
(**A**) SRD values are, on average, higher for 2-class classification scenarios (farther from the reference), meaning that there is a greater degree of disagreement between the performance metrics in this case, highlighting the importance for their informed selection and application during model evaluation. The difference is even more pronounced if the dataset is imbalanced. (**B**) Most of the performance metrics are quite robust in multiclass scenarios, while in 2-class cases, the balanced or imbalanced datasets have a much greater effect on model ranking. (Normalized SRD values are shown always on the Y axis. The markers denote average values, and the vertical lines denote 95% confidence intervals.)

If we break down the results according to the 2-class vs. multiclass, and balanced vs. imbalanced cases, we can see how these factors are influencing the SRD values (Figure 4A). Moreover, by combining these factors with the third factor (performance metrics), we can highlight which performance metrics are most sensitive to the specific classification scenario (Figure 4B).

In multiclass cases, the SRD values are lower, which indicates more consistent model ranking by the various performance metrics. Differences are greater in the case of 2-class classification (Figure 4A), where model ranking is significantly less consistent in the case of imbalanced datasets (which is the most common case in virtual screening tasks!). Figure 4B highlights the performance parameters that are the least consistent between the balanced and imbalanced datasets; these include BEDROC values, average precision (AP), sensitivity (or true positive rate, TPR), the Matthews correlation coefficient (MCC), accuracy (ACC) and balanced accuracy (BACC) as well (!), among others. By comparison, two less-known metrics, markedness (MK) and the diagnostic odds ratio (DOR) are among the most consistent options.

**Figure 5:**
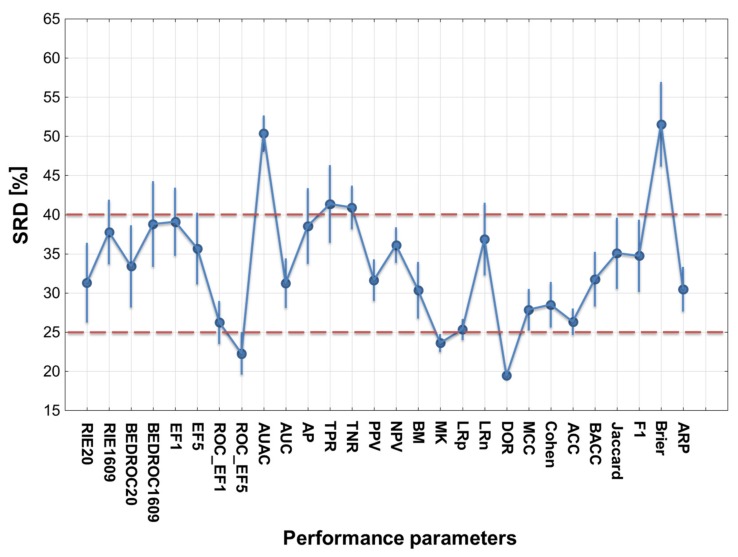
ANOVA result of the performance parameters based on the SRD (%) values. Arbitrary dotted lines denote the classification of the performance parameters into good, medium and not recommended categories.

Focusing only on the performance parameters, the results of the four SRD runs are merged in Figure 5, showing the average SRD values for each performance parameter. If we define two separation thresholds based on the observations, it can be concluded that the most consistent performance parameters are the diagnostic odds ratio (DOR), the ROC enrichment factor at 5% (ROC_EF5) and the markedness (MK). Accuracy is also close the most consistent ones, but interestingly, its balanced analog (BACC) seems to be somewhat worse. At the other end of the scale, area under the accumulation curve (AUAC) and the Brier score loss are not recommended.

**Figure 6:**
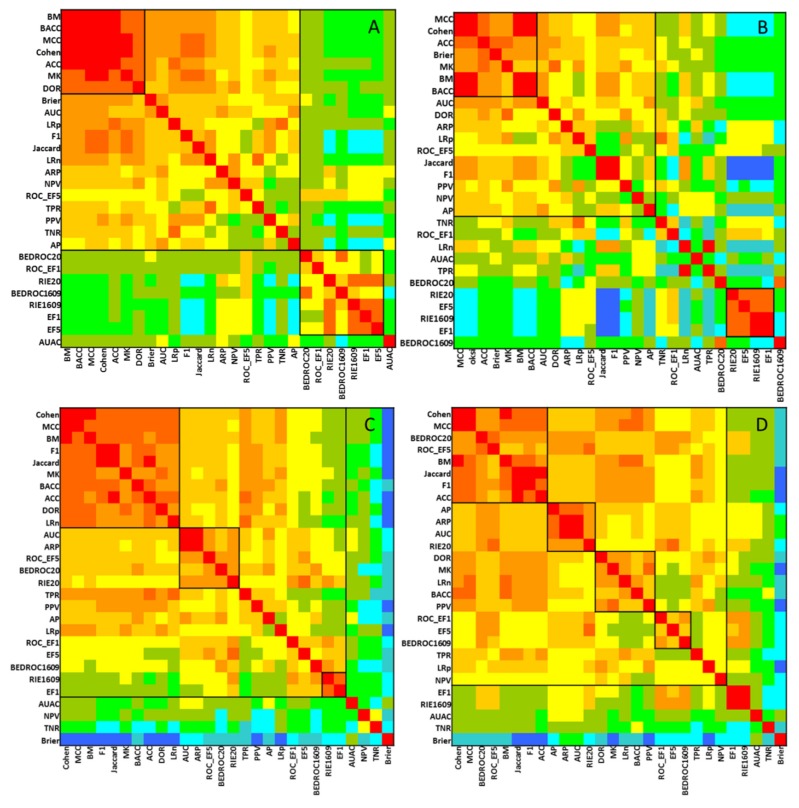
Results of the SRD-COVAT method: 2-class classification with balanced (**A**) and imbalanced (**B**) classes; and multiclass classification with balanced (**C**) and imbalanced (**D**) classes. Clusters of similarly behaving performance parameters are separated with black lines (squares) on the plot based on visual inspection.

The performance parameters were also compared with each other in a pairwise manner on SRD heatmaps (Comparison with One Variable at a Time, COVAT [10]). With this method, clusters of similarly behaving performance parameters can be detected along the diagonal of the heatmap. (The rows and columns of the matrix are reordered in the increasing order of the row-wise average of the SRD values from each run, with different performance metrics as the reference vector.) The heatmaps are included in Figure 6.

The heatmap is arranged in the increasing order of row-wise (and column-wise) sum of SRD scores, with the smallest sums corresponding to the most consistent performance parameters on average. This analysis provides information about the distances between the individual performance metrics (rather than their distance from the consensus-based reference method). The most conserved cluster to be observed consisted of the ACC, BM, Cohen and MCC metrics. 

### 2.2. Statistical Evaluation of Machine Learning Models

**Figure 7:**
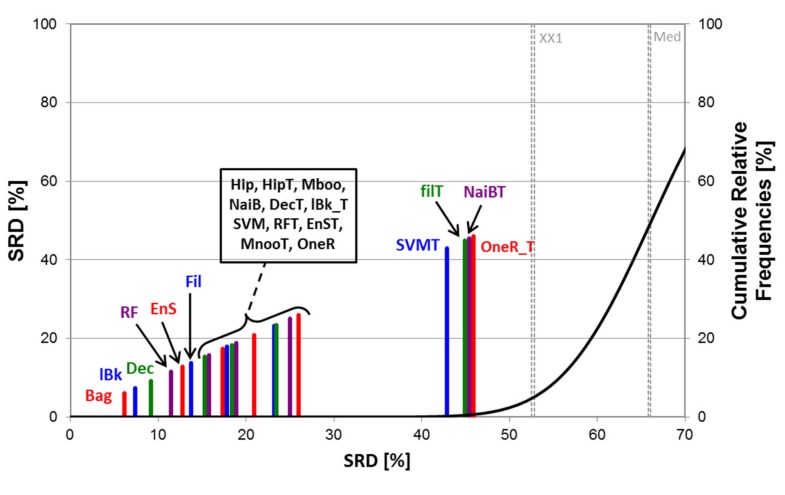
SRD analysis (for the *Daphnia Magna* dataset in the multiclass case, with imbalanced classes). Normalized SRD values are plotted on the X and left Y axes. The abbreviations of the classifiers can be found in Section 3.2. The cumulative relative frequencies (black curve, right Y axis) correspond to the randomization test. The “T” suffix indicates external test validated predictions, the lack thereof indicates cross-validated predictions.

With the same procedure (after transposing the input matrix), machine learning models can also be compared. To briefly recapitulate, the datasets were classified with eleven different machine learning algorithms (Section 3.2), and 28 performance parameters (Section 3.3) were calculated with sevenfold cross-validation and external test validation, as well. Sum of ranking differences (SRD) was applied to the (transposed) input matrices, for balanced vs. imbalanced and for 2-class vs. multiclass cases separately. (In total, 3 × 2 × 2 SRD analyses were carried out with sevenfold cross-validation, one example is included in Figure 7.) A hypothetical best classifier was selected as the reference method; this means row-wise maximums for greater-the-better performance metrics (e.g., AUC values or accuracies) and row-wise minimums for smaller-the-better performance metrics (e.g., negative likelihood ratios or Brier score losses). The ANOVA model was the same as in Equation (1) with one difference: classifiers (11 levels) were applied as factor 3 (instead of the performance parameters).

In Figure 7, the classifiers are tightly grouped and some of them are close to random ranking (however, all of them are significantly better). The relatively small distance from the reference (SRD = 0) suggests that the hypothetical best classifier is well approximated with the bagging (Bag), *k*-nearest neighbor (lBk), and Decorate (Dec) methods.

**Figure 8:**
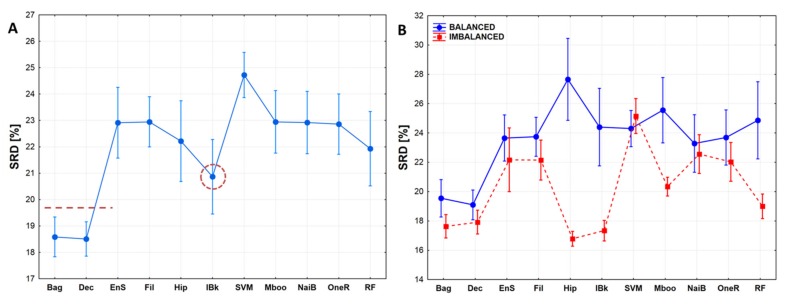
(**A**) Normalized SRD values for the eleven classifiers. Error bars mean 95% confidence intervals. Recommended classifiers are below the dotted line, dotted circle shows an intermediate one. (**B**) Decomposition of the classifiers according to dataset composition (balanced vs. imbalanced classes). Normalized SRD [%] was scaled between 0 and 100.

To allow for more general conclusions, the results of the different SRD runs are summarized in a box-and-whisker plot in Figure 8A. Here, again, Bagging and Decorate seem to be the best, *k*-nearest neighbor has an intermediate position, while others are indistinguishable from each other, and SVM seems to be the worst.

In Figure 8B, characteristic differences can be seen among the classifiers according to the balanced–imbalanced design. Some classifiers, such as Bagging, Decorate, Support vector machine and Naïve Bayes are not sensitive to dataset composition, whereas others, e.g., hyperpipes (hip), *k*-nearest neighbors (lBk), random forest (RF) are highly sensitive. This highlights that the best (or suitable) classifiers can only be selected if the dataset composition is also considered. Interestingly, most machine learning algorithms approximate the ideal reference method more in the case of imbalanced datasets.

## 3. Methods 

### 3.1. Datasets

Three toxicity case studies were used for the multi-level analysis: i) case study 1 contained the 96-hour 50% lethal concentration (LC50) values for *fathead minnow* (Ecotox); ii) case study 2 contained the 48-hour 50% lethal concentration (LC50) values for *daphnia magna* (Ecotox); and iii) case study 3 contained the oral rat 50% lethal dose (LD50) values (TOXNET) [11].

In all three cases, the toxicity categories (aquatic and acute) were determined based on the Globally Harmonized System of Classification and Labelling of Chemicals (GHS). Four category groups were examined (including non-toxic) in the case of aquatic toxicity data and six groups (including non-toxic) in the case of acute oral toxicity data [12]. The dataset compositions are summarized briefly in Figure 2A, and in more detail in Table 1.

Descriptor generation was carried out with DRAGON 7.0 (Kode Cheminformatics). In total, 3839 descriptors were generated (1D and 2D descriptors). Correlated variables (above 0.997) and near constant variables (standard deviation below 0.001) were excluded from further analysis [13]. Training and test splits were applied in the same way as in the original databases.

For 2-class classification, the two extreme classes, i.e., the non-toxic and the most toxic classes of the same datasets were used. For both 2-class and multiclass cases, balanced datasets were prepared by keeping *n* randomly selected compounds for each class, where *n* is the number of compounds in the smallest class.

### 3.2. Machine Learning Algorithms

The machine learning algorithms in the WEKA node package of the KNIME Analytics Platform 3.7.1 (KNIME GmbH, Konstanz, Germany) were applied in this study. Eleven machine learning algorithms were tested: these are summarized in Table 2.

The machine learning models were applied for each combination of balanced vs. imbalanced distribution, and 2-class vs. multiclass classification, in order to examine the effect of these parameters on the results. During each model building phase, fivefold randomized cross-validation with stratified sampling was used.

### 3.3. Performance Metrics

The models were evaluated with 28 different performance metrics. We calculated these metrics for the cross-validation and the external test sets as well. The classifiers provided probability values for each molecule and each class: predicted class labels were assigned based on the class with the highest probability value (for both 2-class and multiclass cases).

**Figure 9:**
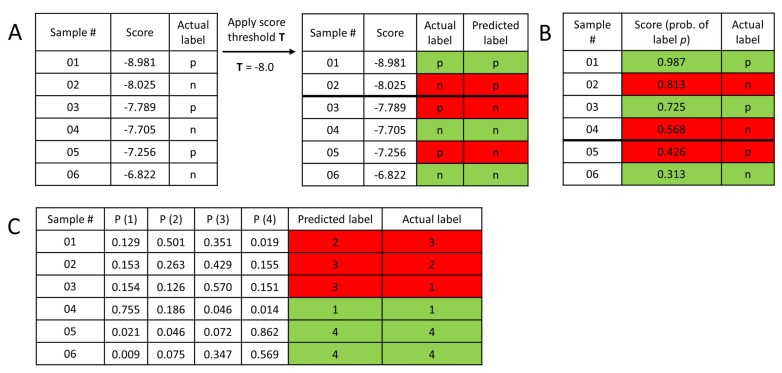
Mock datasets to showcase common classification scenarios. (**A**) In structure-based virtual screening, a docking score is commonly used as a rough estimator of the free energy of binding between ligand and protein (the smaller the better). Predicting a ligand to be active/positive requires setting a threshold value of the docking score (**T**): each ligand with a better score will be considered a predicted active/positive. (**B**) In 2-class classification, machine learning methods typically output probability values for each sample, for belonging to the positive class. A probability value of 0.5 or higher is a natural choice to assign the samples into the positive class. Naturally, other choices can be applied as well: in the above example, setting the threshold value to either 0.6 or 0.4 would reduce the number of misclassified samples by one. (**C**) In multiclass classification, the most straightforward option is to assign each sample to the class with the highest predicted probability. (Green: correct, red: incorrect classification.).

Most performance metrics use the confusion matrix of the observations (number of samples from actual class A, predicted to class B, for each combination of the available classes) in some way. As the simplest example, the confusion matrix for 2-class classification problems can be seen in Table 3. To summarize the most typical classification scenarios in drug discovery/cheminformatics, the reader is referred to Figure 9.

In the present work, 28 performance metrics were collected and applied, most of which were introduced specifically for 2-class classification. To generalize these to multiclass scenarios, weighted averages of each such performance metric were calculated after evaluating them *k* times: each time labelling one of the classes as the positive class, while labelling the rest as the negative class (*k*: number of classes). Metrics that are readily generalized to more classes are highlighted in their description in Table 5.

The metrics can be grouped according to their properties in 2-class classification; they can either operate with one specific classifier threshold (we term these local performance metrics here, see Table 4 and Table 5) or encompass the whole range of possible classifier thresholds (global metrics, Table 6). Local metrics are further divided into one-sided (Table 4) and two-sided (Table 5). One-sided metrics account for exactly two cells of the 2-class confusion matrix (either a row or a column) and always have a complementary metric, providing the same information on a reversed scale. By contrast, two-sided metrics account for more than two cells of the 2-class confusion matrix, and are often derived from one-sided metrics (see for example the definitions of the F1 score, or markedness).

### 3.4. Statistical Evaluation

Sum of ranking differences (SRD), a robust and novel statistical method was used to compare the performance metrics and machine learning methods [33]. The basic concept of this method is the following: (i) the input dataset should contain the objects (here, molecules) in the rows and the methods in the columns (here, performance parameters or machine learning classifiers); (ii) a reference vector (benchmark, or row-wise data fusion, e.g., average, minimum or maximum) should be defined and added as the last column of the matrix: this corresponds to an ideal reference method; (iii) the methods (columns) are ranked one-by-one in increasing magnitude (including the reference column); (iv) the differences between the ranks of each sample between each method and the reference vector are calculated; and finally (v) these differences are summed for each method: these sums are called SRD values, with the smaller value being the better (closer to the ideal reference method). The SRD method is validated with *n*-fold cross-validation (randomized bootstrap version) and a randomization test. The latter can help to identify those methods which are not distinguished from random ranking. Normalized SRD values are also calculated, because this way, the results of different SRD calculations are comparable. A detailed workflow for the better understanding of the procedure can be found in our recent work [34].

The cross-validated, normalized SRD values were used for further analysis of different algorithms and performance parameters. Factorial ANOVA was used for this task, where the effect of three different factors were decomposed: (i) dataset composition (balanced vs. imbalanced); (ii) classification types (2-class, multiclass); (iii) performance parameters (28 levels). Similarly, for comparing machine learning methods, the following factors were considered: (i) dataset composition (balanced vs. imbalanced); (ii) classification types (2-class, multiclass); (iii) classifiers (11 levels). A complete workflow of the process from descriptor generation to ANOVA is included in Figure 1 (Introduction).

For comparing the performance metrics, the SRD-COVAT approach was also applied [10]: briefly, each performance metric is selected as the reference vector, in turn, and the results are summarized in a heatmap format. An absolute coloring scheme was applied for the heatmaps, because we wanted to compare the four different combinations of balanced vs. imbalanced datasets and 2-class vs. multiclass scenarios (see Figure 2B and Figure 6). For more information about the SRD algorithm, with downloadable VBA scripts, consult our website: http://aki.ttk.mta.hu/srd.

## 4. Conclusions

A statistical comparison of 28 classification performance metrics and 11 machine learning classifiers was carried out on three toxicity datasets, in 2-class and multiclass classification scenarios, with balanced and imbalanced dataset compositions.

Our analysis highlighted two lesser-known performance metrics, the diagnostic odds ratio (DOR), and markedness (MK) as the best options, along with the ROC enrichment factor at 5% (ROC_EF5%). By contrast, the following performance parameters are not recommended for model selection: Brier score loss, Area under the accumulation curve (AUAC), true negative rate (TNR) and true positive rate/sensitivity (TPR). DOR and MK are also among the least sensitive metrics to dataset composition. Conversely, BEDROC values, average precision (AP), sensitivity (or true positive rate, TPR), the Matthews correlation coefficient (MCC), accuracy (ACC) and, surprisingly, even balanced accuracy (BACC) are among the most sensitive ones. Most of the performance metrics are sensitive to dataset composition, especially in 2-class classification problems.

From machine learning classifiers, Bagging and Decorate were the best options based on the SRD analysis, while SVM was the weakest, probably due to the non-optimal, automatic selection of the regularization parameters (although, naturally, it is significantly better than random ranking, as well). Bagging, Decorate, Support vector machine and Naïve Bayes are not sensitive to dataset composition, whereas others e.g., hyperpipes (hip), *k*-nearest neighbors (lBk) and random forest (RF) are highly sensitive. Therefore, the best (or suitable) classifiers can only be selected if the dataset composition is also considered.

Sum of ranking differences (SRD), coupled with variance analysis (ANOVA), provides a unique and unambiguous ranking of performance parameters and classifiers, in which even small differences are detected; the methods are comparable in a pair-wise manner as well, with the SRD-COVAT heatmaps.

## Figures and Tables

**Table 1:** Dataset compositions for the three case studies.

			Class 1	Class 2	Class 3	Class 4	Class 5	Class 6
Dataset 1	Balanced	Training	116	116	116	116		
		Test	29	50	48	37		
	Imbalanced	Training	116	166	213	164		
		Test	29	50	48	37		
Dataset 2	Balanced	Training	28	28	28	28		
		Test	8	8	24	15		
	Imbalanced	Training	48	65	58	84		
		Test	8	8	24	15		
Dataset 3	Balanced	Training	199	199	199	199	199	199
		Test	58	132	267	587	291	14
	Imbalanced	Training	199	557	1053	2325	1178	619
		Test	58	132	267	587	291	14

**Table 2:** Summary of the machine learning algorithms. Abbreviations are the names found in the WEKA package. Classification schemes are more general categories (types) of the algorithms. (* Func. is short for “Function”.)

Name (Abbreviation)	Class. Scheme	Details
Naïve Bayes (NaiB)	Bayes	This algorithm is based on the Bayes theorem and the assumption of the independence of all attributes. The samples are examined separately and the individual probability of belonging to a class is calculated for each particular class. Standard options were used in WEKA NäiveBayes node [14].
FilteredClassifier (Fil)	Meta	The algorithm is running an arbitrary classifier on data that has been passed through an arbitrary filter. Attribute selection filter was used with CfsSubset Evaluation and the best first search method [15].
lBk, *k*-nearest neighbour (lBk)	Lazy	One of the simplest algorithms, where the class membership is assigned based on the majority vote of the *k*-nearest neighbours of an instance. Euclidean distance was used as distance measure and *k* = 1 was the number of used neighbours [16].
HyperPipe (Hip)	Misc	Fast and simple algorithm, which is working well with many attributes. The basic idea of the method is the construction of pipes with different pattern of attributes to each class. The samples are monitored and selected to each class based on the pipes and the corresponding class [17].
MultiboostAB (Mboo)	Meta	This algorithm is the modified version of the AdaBoost technique with wagging. The idea of wagging is to assign random weights to the cases in each training set based on Poisson distribution. In this case Decision stump classifier was used. The number of iteration was 10 and the weight threshold was 100. The number of subcommittees was set to 3 [18].
libSVM, library SVM (SVM)	Func.*	Support vector machine can define hyperplane(s) in a higher dimensional space to separate the classes of samples distinctly. The plane should have the maximum margin between data points. Support vectors (points) can maximize the margin of the classifier. Different kernel functions and optimization parameters can be used for the classification task with SVM [19]. In this case radial basis function (RBF) was used as the kernel.
oneR, based on 1-rule, (OneR)	Rule	This algorithm ranks the attributes based on the error rate (on the training set). The basic concept is connected to 1-rules algorithms, where the samples are classified based on a single attribute [20]. Numeric values are treated as continuous ones. In this case, bucket size was 6 (standard) for the discretizing procedure of the attributes.
Bagging (Bag)	Meta	The basic concept of bagging is the creation of different models based on the bootstrapped training sets. The average (or vote) of these multiple versions are used for the prediction of class memberships for each sample [21]. In this case the number of iterations for bagging was set to 10.
Ensemble Selection (EnS)	Meta	It combines several classifier algorithms in the ensemble selection. The average prediction of the models in the ensemble is applied for the class membership determination. The selection of the models is based on an error metric (in our case RMSE). Forward selection was used for the optimization process of the ensemble. Iterations (here, 100) are also carried out such as in the case of Bagging.
Decorate (Dec)	Meta	It is also an ensemble-type algorithm, where the ensembles are constructed directly with diverse hypotheses with the application of additional artificially-constructed training examples to the original one. The classifier is working on the union of the original training and the artificial data (diversity data). The new classifiers are added to the ensemble, if the training error is not increased [22]. Several iterations are carried out to make the prediction stronger. Here, we applied 10 iterations.
Random Forest (RF)	Trees	Random forest is a tree-based method, which can be used for classification and regression problems alike. The basic idea is that it builds many trees and each of them predicts a classification. The final classification is made by a voting of the sequences of trees. The trees are weak predictors, but together they produce an ensemble; with the vote of each tree, the method can make good predictions [23].

**Table 3:** Confusion matrix of observations in 2-class classification.

	Predicted + (PP)	Predicted − (PN)
Actual + (**P**)	True positive (**TP**)	False negative (**FN**)
Actual **−** (**N**)	False positive (**FP**)	True negative (**TN**)

**Table 4:** Local performance metrics for 2-class classification—One-sided.

Name	Alternative Names	Formula	Complementary Metric	Complementary Metric Formula
True positive rate (TPR)	Sensitivity, recall, hit rate	$TPR=\frac{TP}{P}=\frac{TP}{TP+FN}=1-FNR$	False negative rate (FNR), miss rate	$FNR=\frac{FN}{P}=\frac{FN}{TP+FN}=1-TPR$
True negative rate (TNR)	Specificity, selectivity	$TNR=\frac{TN}{N}=\frac{TN}{TN+FP}=1-FPR$	False positive rate (FPR), fall-out	$FPR=\frac{FP}{N}=\frac{FP}{TN+FP}=1-TNR$
Positive predictive value (PPV)	precision	$PPV=\frac{TP}{TP+FP}=1-FDR$	False discovery rate (FDR)	$FDR=\frac{FP}{TP+FP}=1-PPV$
Negative predictive value (NPV)		$NPV=\frac{TN}{TN+FN}=1-FOR$	False omission rate (FOR)	$FOR=\frac{FN}{TN+FN}=1-NPV$

**Table 5:** Local performance metrics for 2-class classification—Two-sided. (*n*: total number of samples, *k*: total number of classes).

Name	Formula	Description
Accuracy (ACC), or Correct classification rate (CC)	$ACC=\frac{TP+TN}{P+N}=\frac{TP+TN}{TP+TN+FP+FN}$ $ACC=\frac{correctly\;predicted}{total}$	Readily generalized to multiple classes. Complementary metric: misclassification rate (or zero-one loss, or Hamming loss).
Balanced accuracy (BACC)	$BACC=\frac{TPR+TNR}{2}$ $BACC=\frac{\sum_{j=1}^{k}\frac{n_{j,corr.}}{n_{j,actual}}}{k}$	Alternative of accuracy for imbalanced datasets. Readily generalized to multiple (k) classes. $n_{j,corr.}$: number of samples correctly predicted into class j $n_{j,actual}$: actual number of samples in class j
F1 score (F1), or F measure	$F=2\times \frac{PPV\times TPR}{PPV+TPR}=\frac{2TP}{2TP+FP+FN}$	Harmonic mean of precision and recall
Matthews correlation coefficient (MCC) [24], φ coefficient (Pearson) [25]	$MCC=\frac{TP\times TN-FP\times FN}{\sqrt{\left(TP+FP\right)\left(TP+FN\right)\left(TN+FP\right)\left(TN+FN\right)}}$ $MCC=\frac{n_{correct}\times n-\sum_{j=1}^{k}n_{j,pred.}\times n_{j,actual}}{\sqrt{\left(n^{2}-\sum_{j=1}^{k}n_{j,pred.}{}^{2}\right)\times }\left(n^{2}-\sum_{j=1}^{k}n_{j,actual}{}^{2}\right)}$	Readily generalized to multiple classes. $n_{j,pred.}$: number of samples predicted into class j $n_{j,actual}$: actual number of samples in class j $n_{correct}$: total no. of correctly predicted samples n: total no. of samples
Bookmaker informedness (BM), or Informedness [26]	BM=TPR+TNR−1	
Markedness (MK) [26]	MK=PPV+NPV−1	
Positive likelihood ratio (LR+)	$LR+\;=\;\frac{TPR}{FPR}$	
Negative likelihood ratio (LR−)	$LR-\;=\;\frac{FNR}{TNR}$	
Diagnostic odds ratio (DOR)	$DOR\;=\;\frac{LR+}{LR-}$	
Enrichment factor (EF)	$EF_{x\%}=\frac{\frac{TP}{PP}}{\frac{P}{P+N}}$	Ratio of true positives in the top x% of the predictions, divided by ratio of positives in the whole dataset.
ROC enrichment (ROC_EF) [27]	$ROC\_EF_{x\%}=\frac{TPR}{FPR_{x\%}}=\frac{TPR}{x}$	Ratio of TPR and FPR at a fixed FPR value (x). Independent of dataset composition.
Cohen’s kappa [28]	$\kappa =\frac{ACC-baseline}{1-baseline}$ $baseline=\frac{\left(PP\times P\right)+\left(PN\times N\right)}{{\left(P+N\right)}^{2}}$ $baseline=\overset{k}{\underset{j=1}{\sum }}\frac{n_{j,pred.}\times n_{j,actual}}{n^{2}}$	Readily generalized to multiple classes. baseline corresponds to the random agreement probability. $n_{j,pred.}$: number of samples predicted into class j $n_{j,actual}$: actual number of samples in class j n: total no. of samples
Jaccard score (J)	$J=\frac{TP}{TP+FN+FP}=\frac{TP}{P+FP}$	Jaccard-Tanimoto similarity between the sets of predicted and actual (true) labels for the complete set of samples.
Brier score loss (B)	$B=\frac{1}{n}\overset{n}{\underset{i=1}{\sum }}\overset{k}{\underset{j=1}{\sum }}{\left(f_{i,j}-o_{i,j}\right)}^{2}$	Readily generalized to multiple classes. $f_{i,j}$ is the predicted probability of sample i belonging to class j, while $o_{i,j}$ is the actual outcome (0 or 1). Requires predicted probability values for each class. The smaller the better.
Robust initial enhancement (RIE) [29]	$RIE=\frac{\sum_{i=1}^{P}e^{-\alpha r_{i}/n}}{\sum_{i=1}^{P}e^{-\alpha r_{i}/n}{}_{r}}$	$r_{i}$ is the rank of positive sample i in the ordered list of samples and α is a parameter that defines the exponential weight. The denominator corresponds to the average sum of the exponential when P positives are uniformly distributed in the ordered list containing n samples.

**Table 6:** Global performance metrics for 2-class classification.

Name	Formula	Description
Area under the ROC curve (AUC) [30]	Area under the TPR-FPR curve	Probability that a randomly selected positive sample will be ranked before a randomly selected negative.
Area under the accumulation curve (AUAC)	Area under the TPR-score (or TPR-rank) curve	If the ranks are normalized, then 0 ≤ AUAC ≤ 1 Probability that a randomly selected positive will be ranked before a randomly selected sample from a uniform distribution.
Average precision (AP)	Area under the precision-recall (PPV-TPR) curve	
Boltzmann-enhanced discrimination of receiver operating characteristic (BEDROC) [31]	$BEDROC=\frac{RIE-RIE_{min}}{RIE_{max}-RIE_{min}}$	See the definition of RIE above, α is a parameter that defines the exponential weight. 0 ≤ BEDROC ≤ 1 BEDROC is an analog of AUC that assigns an (exponentially) greater weight to high-ranked samples, thus tackling the “early recognition problem”.
Average rank (position) of actives (positives) (r) [32]	$r=\frac{1}{P\times \left(P+N\right)}\overset{P}{\underset{i=1}{\sum }}r_{i}$	$r_{i}$ is the rank of positive sample i in the total ranked list of samples. The smaller the better.
